# Longitudinal alterations of the cisternal segment of trigeminal nerve and brain pain-matrix regions in patients with trigeminal neuralgia before and after treatment

**DOI:** 10.1186/s12868-021-00681-w

**Published:** 2021-12-11

**Authors:** Tai-Yuan Chen, Ching-Chung Ko, Te-Chang Wu, Li-Ching Lin, Yun-Ju Shih, Yi-Chieh Hung, Ming-Chung Chou

**Affiliations:** 1 Department of Radiology, Chi-Mei Medical Center, Tainan, Taiwan; 2 Graduate Institute of Medical Sciences, Chang Jung Christian University, Tainan, Taiwan; 3 Department of Health and Nutrition, Chia Nan University of Pharmacy and Science, Tainan, Taiwan; 4 Department of Medical Sciences Industry, Chang Jung Christian University, Tainan, Taiwan; 5 Department of Biomedical Imaging and Radiological Sciences, National Yang-Ming University, Taipei, Taiwan; 6 Department of Radiation Oncology, Chi-Mei Medical Center, Tainan, Taiwan; 7 Division of Neurosurgery, Departments of Surgery, Chi-Mei Medical Center, Tainan, Taiwan; 8 Department of Recreation and Healthcare Management, Chia Nan University of Pharmacy and Science, Tainan, Taiwan; 9 Department of Medical Imaging and Radiological Sciences, College of Health Sciences, Kaohsiung Medical University, 100, Shih-Chuan 1st Rd, Kaohsiung, 80708 Taiwan; 10 Department of Medical Research, Kaohsiung Medical University Hospital, Kaohsiung, Taiwan; 11 Center for Big Data Research, Kaohsiung Medical University, Kaohsiung, Taiwan

**Keywords:** Trigeminal neuralgia, Trigeminal nerve, Pain-matrix regions, RESOLVE DTI, T2-SPACE VBM

## Abstract

**Background:**

Trigeminal neuralgia (TN) is the most common type of chronic neuropathic facial pain, but the etiology and pathophysiological mechanisms after treatment are still not well understood. The purpose of this study was to investigate the longitudinal changes of the cisternal segment of the trigeminal nerve and brain pain-related regions in patients with TN before and after treatment using readout segmentation of long variable echo-train (RESOLVE) diffusion tensor imaging (DTI) and transverse relaxation (T2)-weighted sampling perfection with application-optimized contrast at different flip angle evolutions (T2-SPACE).

**Methods:**

Twelve patients with TN and four healthy controls were enrolled in this study. All patients underwent assessment of the visual analog scale (VAS), and acquisition of RESOLVE DTI and T2-SPACE images before and at 1, 6, and 12 months after treatments. Regions-of-interest were placed on the bilateral anterior, middle, and posterior parts of the cisternal segment of the trigeminal nerve, the bilateral root entry zone (REZ), bilateral nuclear zone, and the center of pontocerebellar tracts, respectively. Voxel-based morphometry (VBM) analysis was conducted with T2-SPACE images, and gray matter volumes (GMV) were measured from brain pain-matrix regions.

**Results:**

The results demonstrated that the VAS scores, the axial diffusivity of the middle part of the affected cisternal trigeminal nerve, the fractional anisotropy of the bilateral nuclear zones, and the mean diffusivity of the center of pontocerebellar tract significantly changed over time before and after treatment. The changes of GMV in the pain-matrix regions exhibited similar trends to the VAS before and after treatment.

**Conclusion:**

We conclude that magnetic resonance imaging with RESOLVE DTI and VBM with T2-SPACE images were helpful in the understanding of the pathophysiological mechanisms in patients with TN before and after treatment.

## Introduction

Trigeminal neuralgia (TN) is by far the most common type of chronic neuropathic facial pain [1] and is characterized by intermittent attacks of severe, sharp, burning or electric shock-like unilateral pain along the distribution of the trigeminal nerve branches [2]. However, the etiology and pathophysiological mechanisms of TN remain not well understood. A well-established etiological factor is the neurovascular compression (NVC) of the trigeminal nerve at the root entry zone (REZ) [3–5], where focal axonal demyelination is believed to occur at the point of contact [6, 7]. Therefore, the surgical treatments, including Gamma/Cyber Knife radiosurgery (CKRS), microvascular decompression surgery, and percutaneous trigeminal rhizotomy, have been previously performed for patients with TN [8, 9].

Advances in neuroimaging techniques have identified key neuroanatomical signatures for diagnosis of neurological disorders. It is well known that magnetic resonance imaging (MRI) with diffusion tensor imaging (DTI) is capable of depicting microstructural changes of the trigeminal nerve [10]. With the application of readout segmentation of long variable echo-train (RESOLVE) DTI and parallel imaging [11, 12], susceptibility distortions could be reduced substantially to analyze more accurately subtle changes involving the symptomatic cisternal segment of trigeminal nerve, REZ, and the nuclear zone.

In addition, high-resolution longitudinal relaxation (T1)-weighted images were previously utilized to detect whole-brain gray matter (GM) changes using voxel-based morphometric (VBM) analysis. Given that the evaluation of the brain's GM could provide insights into disease mechanisms, it is helpful to use VBM analysis in the detection of GM abnormalities in patients with neuralgia. Previously, brain GM abnormalities have been identified in patients with chronic pain [13]. The most common locations of GM abnormalities include pain-processing brain regions, such as the prefrontal cortex (PFC), insula, anterior cingulate cortex (ACC) and mid-cingulate cortex, thalamus, primary and secondary somatosensory cortices (S1, S2), basal ganglia, amygdala, and brainstem [13]. These abnormalities are commonly found for many types of chronic pain, including those that affect the trigeminal system, such as migraines [14], trigeminal neuropathic pain [15, 16], and temporomandibular disorders [17]. Given that some abnormalities are common across most chronic pains, such as cortical thinning in the anterior insula, cingulate cortex, and dorsolateral PFC [13], the changes of these areas may likely reflect pain chronicity and negative effects in pain modulation [18]. In a previous TN study, the reductions of the volumes of the ACC and the superior temporal gyrus (STG)/middle temporal gyrus were documented [19]. It was also considered that the GM volume (GMV) of the STG decreased as the duration of disease increased. Such MR-detectable brain structural alterations may reflect changes in the neuronal size or number, synaptogenesis, dendritic branching, axonal sprouting, synaptic pruning, neuronal cell death, alterations in vasculature, and the sizes or numbers of glial cells [20, 21].

Moreover, transverse relaxation (T2)-weighted sampling with application-optimized contrast with different flip angle evolution (T2-SPACE) images were used to evaluate cortical/subcortical brain GM based on VBM analyses [22]. However, T2-SPACE images have not been previously utilized to investigate longitudinal brain changes in TN subjects after surgeries. As the surgical treatments could relieve pain symptoms in patients, it is of importance to understand how the cisternal segment of the trigeminal nerve and brain GM structures were changed after the treatments. Therefore, the purposes of this study were to evaluate longitudinal diffusion changes of the cisternal segment of trigeminal nerves using RESOLVE DTI technique and to assess longitudinal brain GM differences using T2-SPACE VBM analysis in TN patients after CKRS or radiofrequency ablation (RFA).

## Materials and methods

### Patients

The study received institutional approval from the Human Research Ethics Committee of our hospital (Institutional Review Board 10603-003), and written informed consents were obtained from all participants. We enrolled 12 patients with refractory TN that lasted for more than 2 years, who received CKRS or RFA treatments from January 2016 to December 2018. In addition, four age- and sex-matched healthy controls (2M/2F, age = 63.3 ± 4.4 years) were enrolled for comparisons.

### Data acquisition

After providing informed consent, all subjects underwent pain intensity assessments of the pain intensity and the severity of neuralgia using the visual analog scale (VAS). MRI scans were also conducted to exclude the possibility of occupying lesions. In this study, MRI was conducted on a 1.5-T scanner (MAGNETOM Aera, Siemens Medical Systems, Erlangen, Germany) equipped with a 24-channel phased-array head coil. The standard MRI protocol included the following: axial T1-weighted imaging, T2-weighted imaging, fluid attenuated inversion recovery, T2-weighted gradient-recalled echo imaging, MR angiography, and single-shot spin-echo echo-planar diffusion-weighted imaging. In addition, during the same imaging session and follow-up sessions at 1, 6, and 12 months after treatment, all subjects underwent three-dimensional (3D), whole-brain, high-resolution T2-SPACE imaging (TR/TE = 2200/276 ms, echo train length = 150, number of slice = 192, matrix size = 256 × 256, voxel size = 1 × 1 × 1 mm^3^), in which an imaging plane parallel to cisternal segments of trigeminal nerves was placed to acquire both single-shot DTI (TR/TE = 6000/87 ms, number of slice = 21, slice thickness = 2 mm, no gap, FOV = 22 cm, matrix size = 128 × 128, number of excitation = 1, acceleration factor = 2, b-values = 0 and 1000 s/mm^2^) and RESOLVE DTI (TR/TE = 3330/75 ms, number of slice = 21, slice thickness = 2 mm, no gap, FOV = 22 cm, matrix size = 128 × 128, number of excitation = 1, number of segment = 4, acceleration factor = 2, b-values = 0 and 1000 s/mm^2^) with 30 diffusion directions.

### Image processing and analysis

**Fig. 1:**
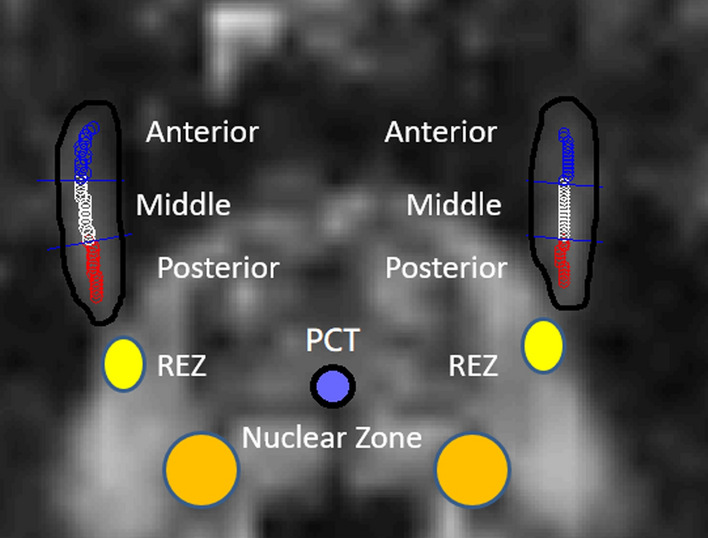
Illustration of placements of ROI in the anterior, middle, and posterior parts of the cisternal trigeminal nerve; root entry zone (REZ); nuclear zone; and center of pontocerebellar tracts (PCT)

All imaging data were transferred to a stand-alone workstation and post-processed with FSL (FMRIB Software Library, Oxford, UK) and MATLAB (MathWorks Inc., Natick, MA, USA). First, the DTI data were motion-corrected using rigid-body registration, and the diffusion directions were compensated by the rotation matrix. Second, eddy-current distortions were minimized using 12-parameter affine image registration. Third, the fractional anisotropy (FA), axial diffusivity (AD), radial diffusivity (RD), and mean diffusivity (MD) values in each voxel were calculated with the DTIFIT tool. Fourth, the DTI metrics were analyzed in the trigeminal nerve cisternal segment on the affected and contralateral sides based on the selection of a region-of-interest (ROI) with custom-made software that ran on a MATLAB platform that automatically divided the trigeminal nerve cisternal segment into anterior, middle, and posterior parts, as shown in Fig. 1.

Moreover, individual FA maps were spatially transformed to a representative FA map—which used the minimal transformation distance among all patients—using linear affine and nonlinear demon registrations. Subsequently, ROIs were placed on the REZ, nuclear zone, and the center of pontocerebellar tracts (PCT) in the pons, as shown in Fig. 1. Finally, the mean FA, AD, RD, and MD values in these regions were calculated for statistical analysis.

The whole-brain T2-SPACE images were used in VBM analysis to estimate the brain GMV with the Computational Anatomical Toolbox version 12, (CAT12) and Statistical Parametric Mapping version 12 (SPM12) in MATLAB. First, T2-SPACE images were segmented into GM, white matter, and cerebrospinal fluid, spatially normalized with diffeomorphic anatomical registration through exponentiated Lie algebra (DARTEL) to the subject-specific template. The segmented images were then smoothed with an isotropic Gaussian kernel with a full-width-at-half-maximum equal to 8 mm. Subsequently, an automated anatomical labeling brain template was used to separate the brain cortex into 116 regions, and mean GMVs were calculated in the pain-related regions, including the periaqueductal GM (PAG), PFC, posterior cingulate cortex (PCC), ACC, insula, amygdala, thalamus, posterior parietal cortex (PPC), S1, S2, supplementary motor area (SMA), and cerebellum [23].

### Statistical analysis

A Wilcoxon signed-rank test was performed to understand the difference of DTI indices in the cisternal segments of the trigeminal nerve, REZ, nuclear zone, and the center of PCT, and the difference of GMV in pain-related brain regions in healthy controls and patients between the two hemispheres, respectively. The results were considered significant if P < 0.05.

In addition, one-way analysis of variance (ANOVA) was performed to show whether the DTI indices changed along the three parts of the cisternal segments of the trigeminal nerve and whether the DTI indices of the trigeminal nerve, REZ, nuclear zone, center of PCT, the GMV of pain-related brain regions, and the VAS, significantly changed over time before and after treatment, respectively. The results were considered significant if P < 0.05. The post-hoc Wilcoxon signed-rank test was performed to understand the difference between two values, and the difference was significant if corrected P < 0.05 with Bonferroni correction.

Moreover, Pearson's correlational analysis was performed to reveal the relationship between DTI indices of trigeminal nerve, REZ, nuclear zone, the center of PCT, and the GMV of pain-related brain regions. The correlations were considered significant if P < 0.005.

## Results

The mean age of the 12 enrolled patients was 68.9 years, including four men and eight women. All of them had TN unilaterally, with 11 on the right side and 1 on the left side. None of them had definite space-occupying lesions. All patients successfully underwent VAS assessments before and at 1, 6, and 12 months after treatment. However, in the imaging study, although all patients underwent MRI prior to the treatment, six patients did not undergo a follow-up MRI at 1 and 6 months, and eight patients did not undergo a follow-up MRI at 12 months after treatment owing to refusals or dropout. The demographic and clinical characteristics of the enrolled patients are listed in Table 1.

**Table 1:** Demographic characteristics of patients with trigeminal neuralgia (TN) enrolled in this study

Patient	NVC	VAS score				Procedure
		Before Tx	1 month	6 months	12 months	
1	No	7	0	0	0	CKRS
2	Rt. REZ	8	0	0	0	CKRS
3	Lt. REZ	9	1	0	0	RFA
4	Rt. REZ	9	0	9	2	RFA, CKRS
5	Rt. REZ	10	0	0	0	RFA, CKRS
6	Rt. REZ	9	0	0	0	RFA
7	Rt. REZ	9	0	2	4	CKRS, RFA
8	No	10	0	0	0	CKRS
9	Rt. REZ	10	0	0	0	CKRS
10	Rt. REZ	10	0	0	0	RFA
11	Lt. middle part	10	0	0	0	RFA
12	No	10	0	0	0	CKRS

NVC: neurovascular compression; Tx: treatment; REZ: root entry zone; VAS: visual analog scale; CKRS: cyber knife radiosurgery; RFA: radiofrequency ablation

The diagnostic MRI demonstrated that among the 12 patients, eight had NVC on the trigeminal nerve REZ, one had NVC at the middle part of the cisternal segment of trigeminal nerve, and three had no NVC signs. In addition, six patients received RFA, and others received CKRS treatment. However, after treatment, two patients experienced TN relapse at 6 months and received an additional treatment using CKRS or RFA. In the VAS analysis, the results showed that the initial VAS was as high as 9.25 ± 0.97 (10 was the highest score). After 1 month of treatment, the VAS was significantly reduced to 0.08 ± 0.29. However, the VAS was slightly increased to 0.92 ± 2.61 at 6 months but was decreased to 0.50 ± 1.24 at 12 months after treatment. The ANOVA analysis revealed that the VAS significantly changed over time before and after treatment.

### Diffusion changes using RESOLVE DTI technique

**Fig. 2:**
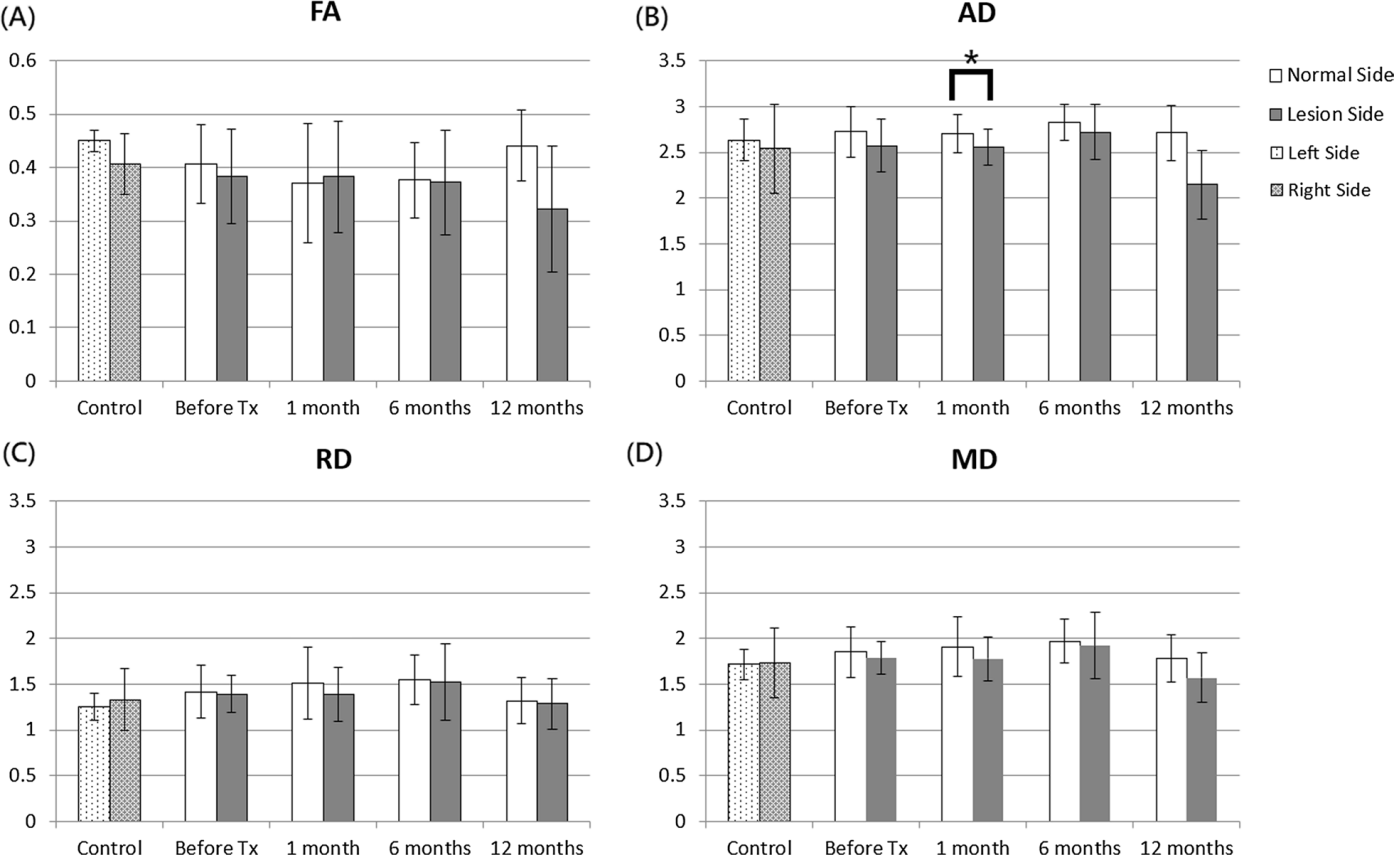
FA, AD, RD, and MD values between affected and contralateral sides in the anterior part of the cisternal trigeminal nerve in healthy controls and patients with TN before and after treatment. The unit for AD, RD, and MD is 10^–3^ mm^2^/s. The asterisks (*) indicate P < 0.05

In patients with TN, the entire cisternal segment of the trigeminal nerve exhibited no significant difference in DTI indices between the affected and contralateral sides before and at 1, 6, and 12 months after treatment. In the anterior part of the cisternal trigeminal nerve, no significant difference of FA, AD, RD, and MD values was noted between the two sides before treatment, but significant AD differences were noted between the two sides at 1 month after treatment (Fig. 2). However, in healthy controls, no significant differences in the DTI indices were noted between the two sides. The ANOVA analysis showed no significant changes in the DTI indices over time in both sides of the anterior part before and after treatment.

**Fig. 3:**
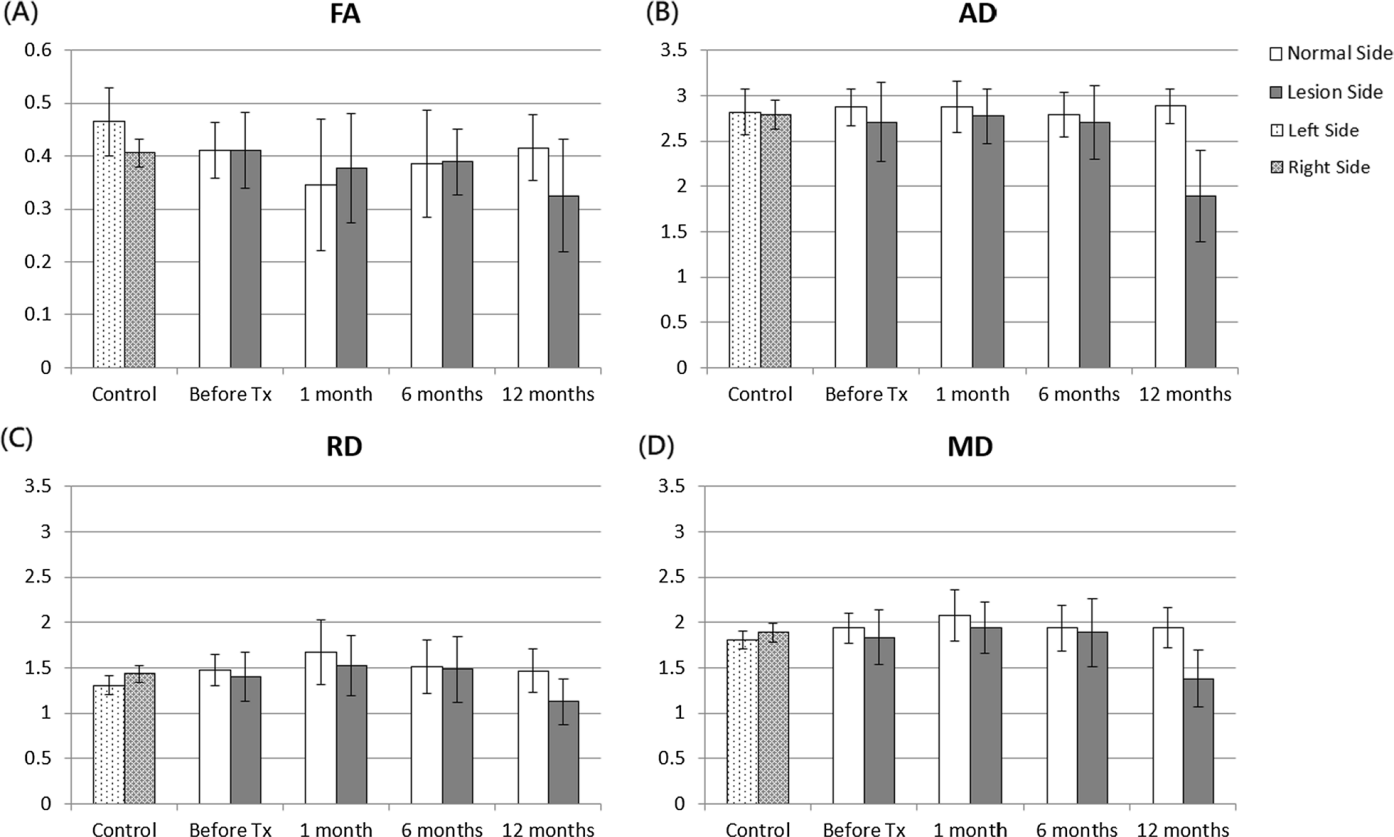
FA, AD, RD, and MD values between affected and contralateral sides in the middle parts of the cisternal trigeminal nerve in healthy controls and patients with trigeminal neuralgia (TN) before and after treatment. The unit for AD, RD, and MD is 10^–3^ mm^2^/s. The asterisks (*) indicate P < 0.05

In the middle part of the cisternal trigeminal nerve, the results yielded no significant differences for FA, AD, RD, and MD values between the two sides before, and at 1, 6, and 12 months after treatment, as shown in Fig. 3. However, in healthy controls, no significant differences were noted in the DTI indices between the two sides. The ANOVA analysis showed no significant changes in the DTI indices over time in both sides of the middle parts before and after treatment.

**Fig. 4:**
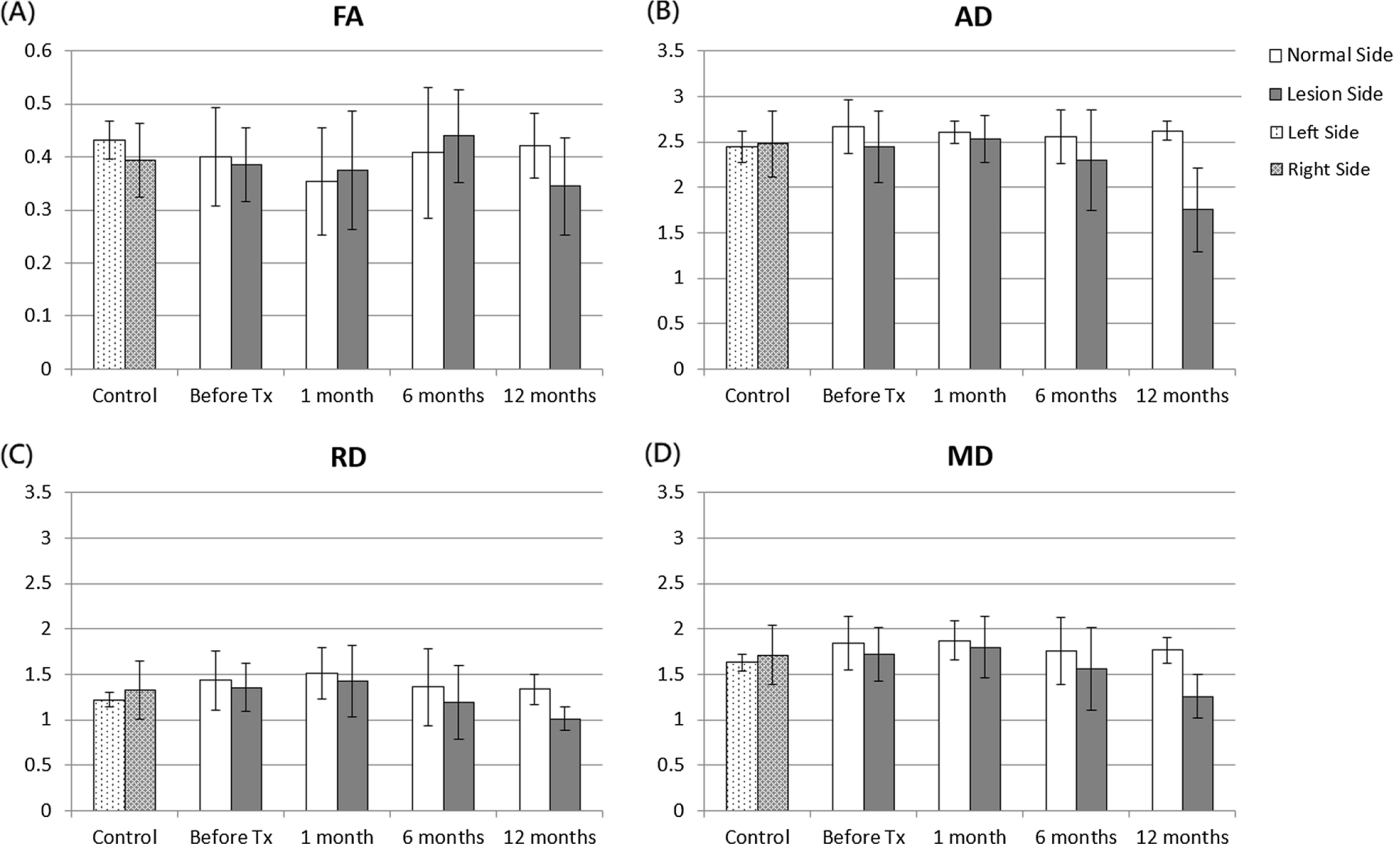
FA, AD, RD, and MD values between affected and contralateral sides in the posterior parts of the cisternal trigeminal nerve in healthy controls and patients with TN before and after treatment. The unit for AD, RD, and MD is 10^–3^ mm^2^/s. The asterisks (*) indicate P < 0.05

In the posterior part of the cisternal trigeminal nerve, no significant difference was noted in FA, AD, RD, and MD values between the two sides in patients with TN before and at 1, 6, and 12 months after treatment, as shown in Fig. 4. In healthy controls, no significant differences were noted in the DTI indices between the two sides. The ANOVA analysis further revealed that only the AD values significantly changed over time (P = 0.034) in the middle part of the cisternal trigeminal nerve of the affected side before and after treatment. However, no significant difference was noted between two time points after Bonferroni correction.

**Fig. 5:**
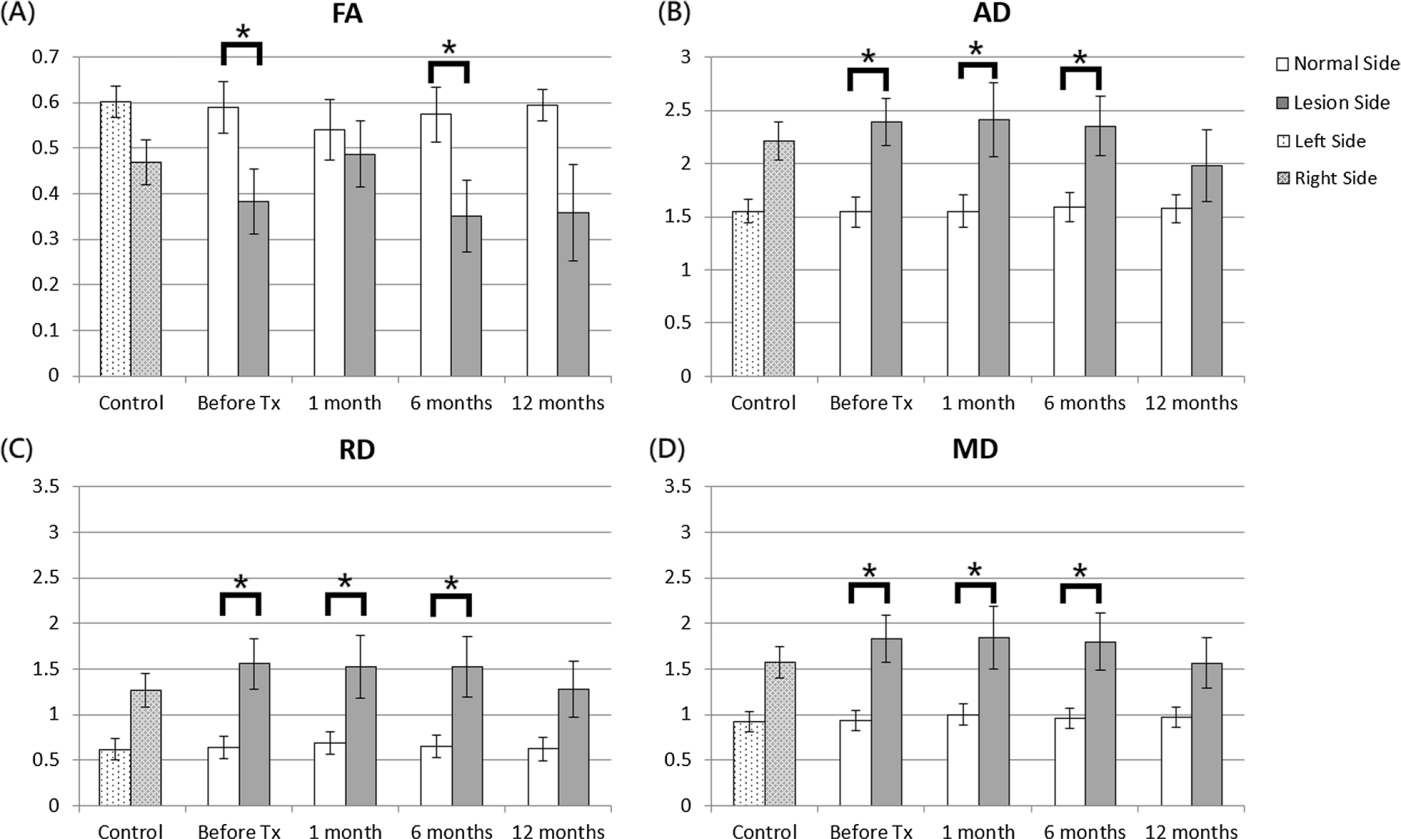
FA, AD, RD, and MD values between affected and contralateral sides in the trigeminal nerve REZ in healthy controls and patients with TN before and after treatment. The unit for AD, RD, and MD is 10^–3^ mm^2^/s. The asterisks (*) indicate P < 0.05

In the REZ, the results yielded significant differences for the FA values between the two sides in the patients with TN before and 6 months after treatment and significant differences for the AD, RD, and MD values before and at 1 and 6 months after treatment, as shown in Fig. 5. In healthy controls, no significant differences in the DTI indices were noted between the two sides. The ANOVA analysis showed no significant changes in the DTI indices over time in both sides before and after treatment.

**Fig. 6:**
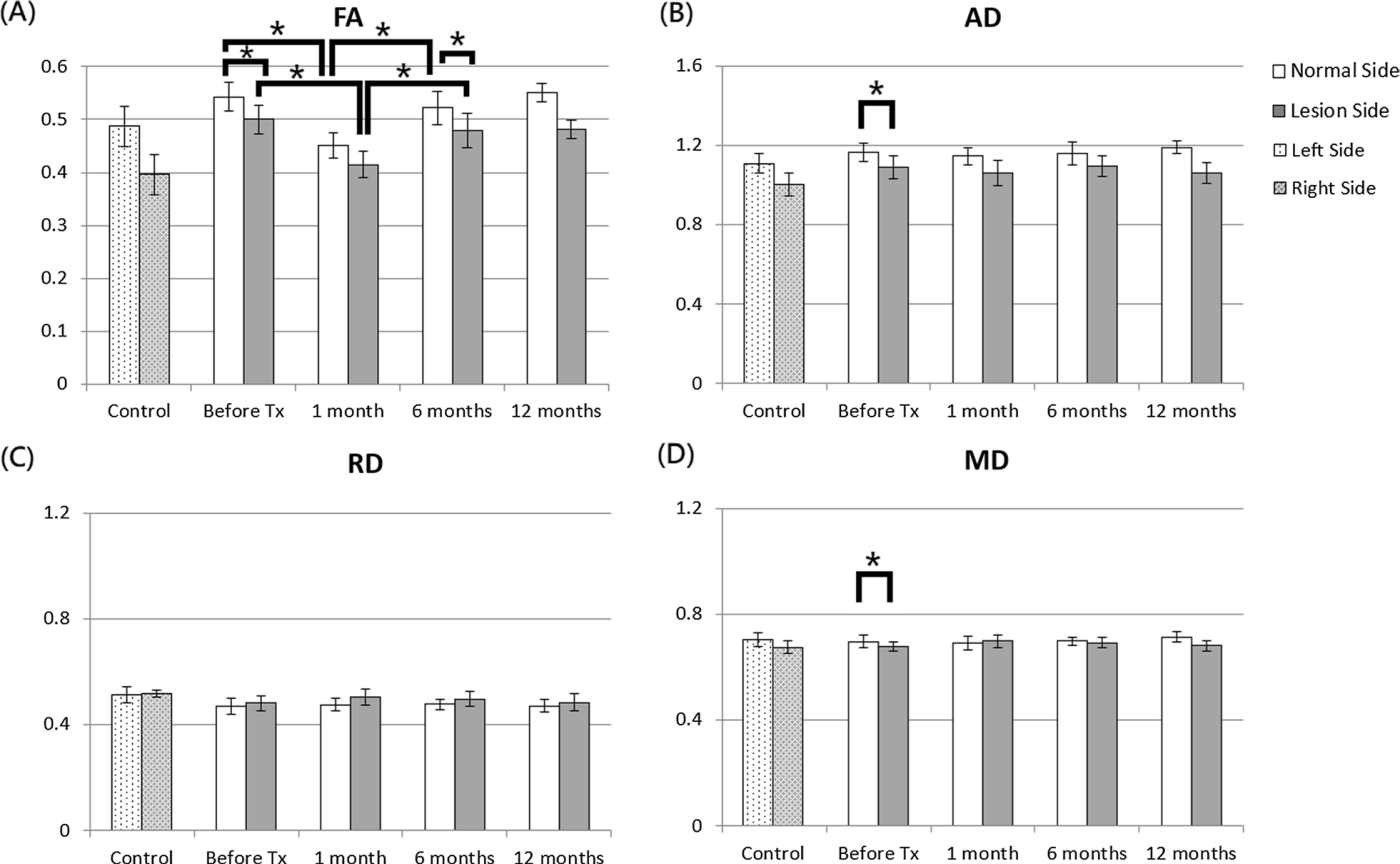
FA, AD, RD, and MD values between affected and contralateral sides in the nuclear zones in healthy controls and patients with TN before and after treatment. The unit for AD, RD, and MD is 10^–3^ mm^2^/s. The asterisks (*) indicate P < 0.05

In the nuclear zone, the results yielded significant differences in the cases of the FA, AD, and MD values between the two sides in patients with TN before treatment and significant FA value differences 6 months after treatment, as shown in Fig. 6. In healthy controls, no significant differences in the DTI indices were noted between the two sides. Additionally, the ANOVA analysis revealed that the FA value significantly changed over time in the nuclear zones of both sides (P = 0.0002 in the normal side, P = 0.0389 in the lesion side) before and after treatment. Moreover, in both sides, the FA value was significantly reduced 1 month after treatment but was significantly increased from 1 to 6 months after treatment.

**Fig. 7:**
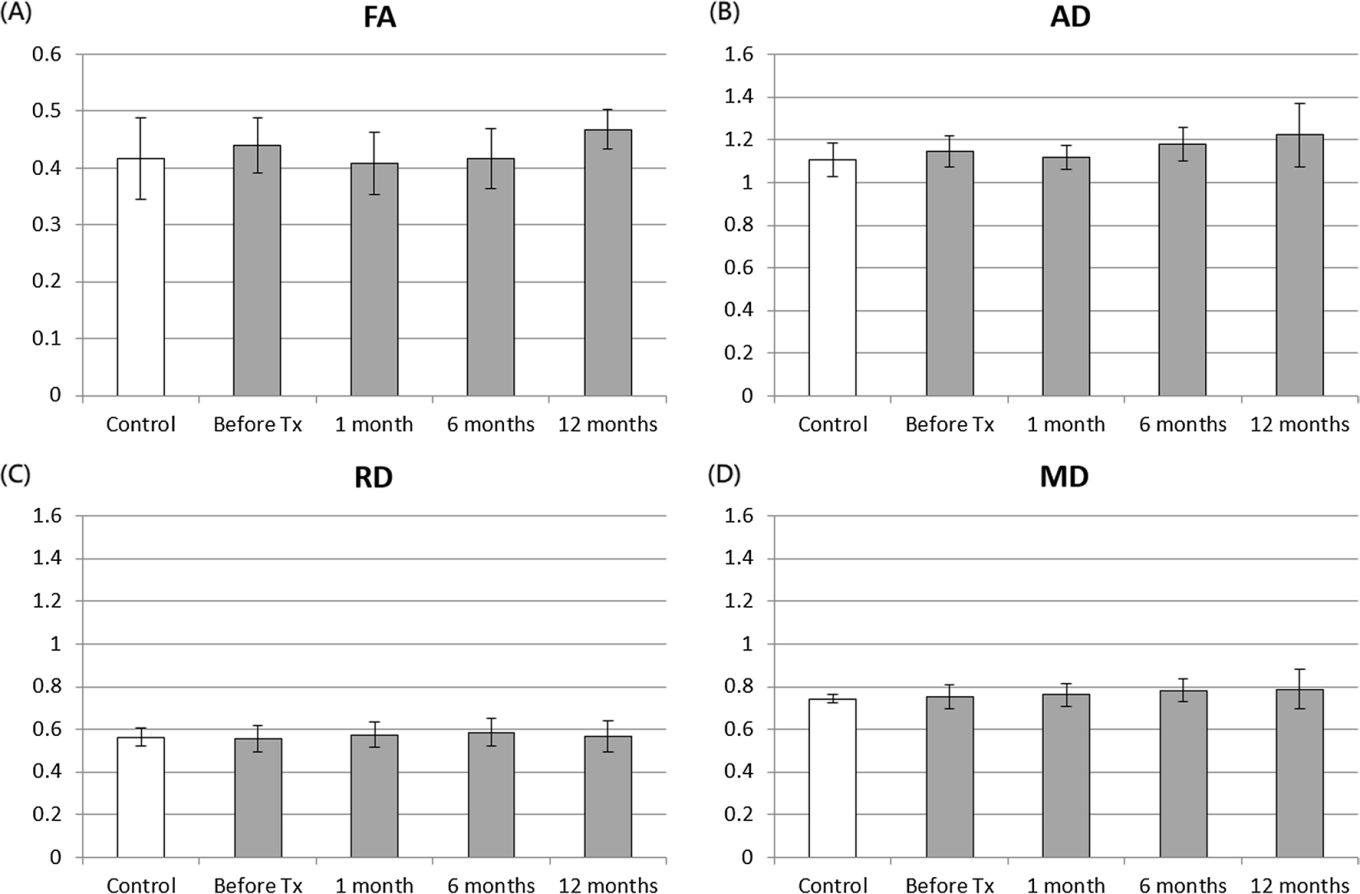
FA, AD, RD, and MD values in the center of PCT in healthy controls and patients with TN before and after treatment. The unit for AD, RD, and MD is 10^–3^ mm^2^/s

In the center of PCT, the FA and AD values were slightly decreased, whereas the RD and MD values were slightly increased in patients with TN 1 month after treatment, as shown in Fig. 7. However, the ANOVA analysis revealed that the MD value significantly increased over time (P = 0.00069) in the center of PCT before and after treatment, but no significant difference was noted between two time points after Bonferroni correction.

### T2-SPACE VBM analysis

**Fig. 8:**
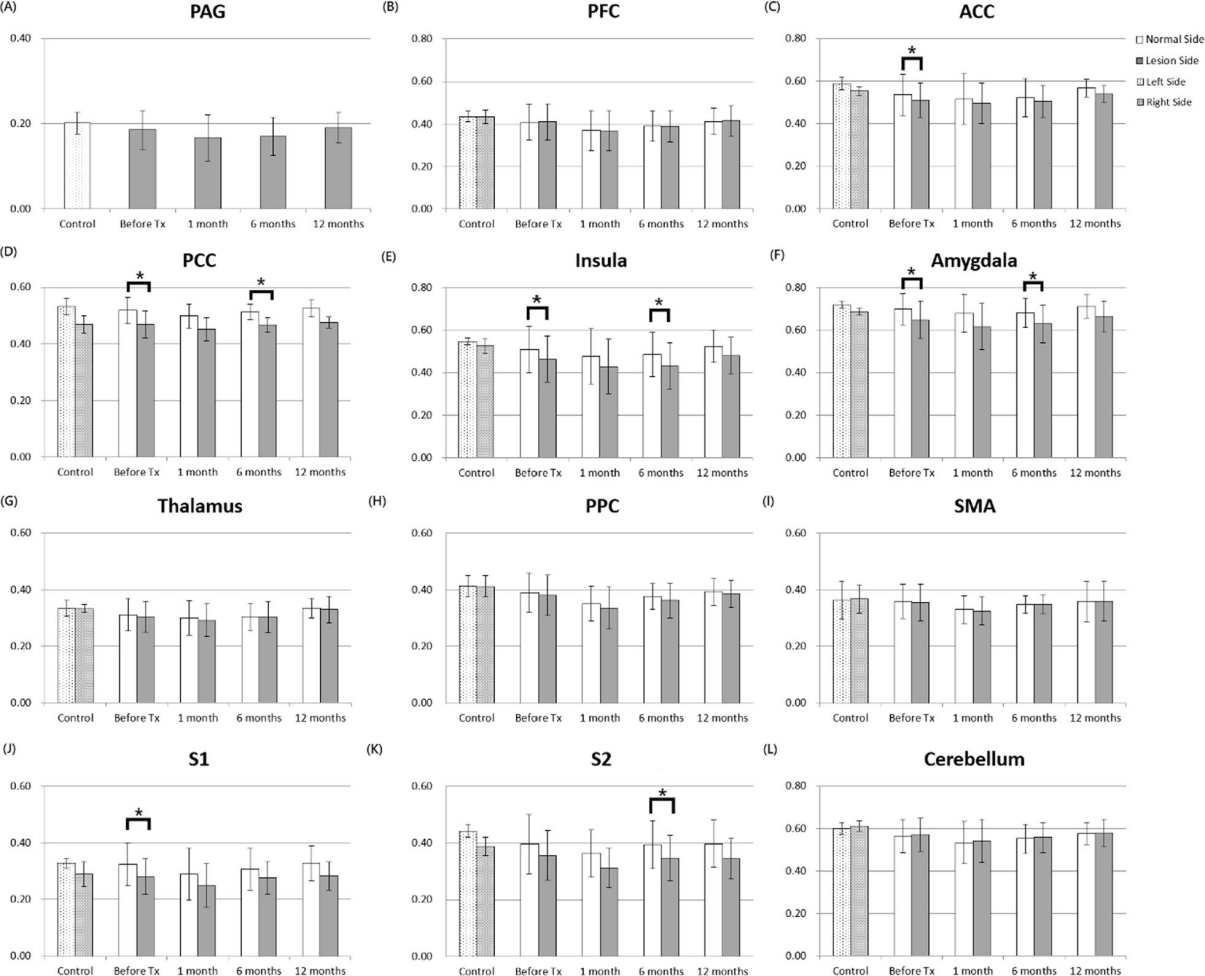
Gray matter volumes (GMV) in the pain-matrix regions in healthy controls and patients with TN before and after treatment. The asterisks (*) indicate P < 0.05

In the VBM analysis, the results revealed that GMV was significantly lower in the affected side compared with the contralateral side in the PCC, ACC, insula, amygdala, and S1 in the patients before treatment. After 1 month of treatment, no significant GMV difference was noted in the pain-related regions between the two hemispheres. However, the GMV was significantly lower in the affected side compared with the contralateral side in the PCC, insula, amygdala, and S2 at 6 months after treatment, but the difference no longer existed in the pain-related regions between the two hemispheres at 12 months after treatment, as shown in Fig. 8. In healthy controls, no significant differences in the GMVs were noted between the two hemispheres. The ANOVA analysis showed that the GMVs did not significantly change over time in the pain-matrix regions of the affected and contralateral sides before and after treatment.

In correlational analysis, the results revealed that before treatment, the FA of the anterior part of the cisternal trigeminal nerve in the affected side was significantly correlated with the GMV of PAG, bilateral PFC, bilateral amygdala, bilateral SMA, bilateral cerebellum, left S1, left PCC, left insula, right PPC, and right ACC. Moreover, the AD of the middle part of the cisternal trigeminal nerve in the affected side was significantly correlated with the GMV of the bilateral PFC, bilateral PPC, and left S2, and the RD and MD values of the middle part of the cisternal trigeminal nerve in the affected side were also significantly correlated with the GMV of the right S2, as shown in Table 2.

**Table 2:** Significant correlation coefficients between DTI indices of cisternal trigeminal nerve and gray matter volume (GMV) of pain-matrix regions in patients with TN before treatment

Region	FA (anterior part of the affected side)	AD (middle part of the affected side)	RD (middle part of the affected side)	MD (middle part of the affected side)
PAG	0.8319	–	–	–
Lt. PFC	0.8454	0.7779	–	–
Rt. PFC	0.8632	0.7916	–	–
Lt. PCC	0.7500	–	–	–
Rt. ACC	0.7560	–	–	–
Lt. amygdala	0.7807	–	–	–
Rt. amygdala	0.8418	–	–	–
Lt. PPC	–	0.8114	–	–
Rt. PPC	0.7889	0.7818	–	–
Lt. SMA	0.8503	–	–	–
Rt. SMA	0.8859	–	–	–
Lt. S1	0.7611	–	–	–
Lt. S2	–	0.8397	–	–
Rt. S2	–	–	0.7658	0.8137
Lt. cerebellum	0.8215	–	–	–
Rt. cerebellum	0.7898	–	–	–

FA: fractional anisotropy; AD: axial diffusivity; RD: radial diffusivity; MD: mean diffusivity; PAG: periaqueductal gray; PFC: prefrontal cortex; PCC: posterior cingulate cortex; ACC: anterior cingulate cortex; SMA: supplementary motor area; S1: primary somatosensory cortex; S2: secondary somatosensory cortex

However, after 1 month of treatment, only the FA of the middle part of the cisternal trigeminal nerve in the affected side yielded a significant correlation with the GMV of PAG (cc = 0.9759). After 6 months of treatment, only the RD of the nuclear zone in the affected side yielded a significant correlation with the GMV in the right S1 (cc = 0.9537). After 12 months of treatment, only the MD of the nuclear zone in the affected side yielded a significant correlation with the GMV in the left thalamus (cc = 0.9957).

## Discussion

To the best of our knowledge, this is the first study that conducted both RESOLVE DTI and 3D T2-SPACE imaging to investigate the longitudinal changes of the cisternal segment of the trigeminal nerve, REZ, nuclear zone, the center of PCT, and brain pain-matrix regions in the patients with TN before and after treatment. Several findings presented herein may help understand the etiology and pathophysiology of TN. First, the trigeminal nerve REZ and nuclear zone exhibited more changes than the cisternal segment of the trigeminal nerve and the GMVs of brain pain-matrix regions before and after treatment. Second, the contralateral REZ and nuclear zone were significantly altered after treatment most likely owing to Wallerian degeneration. Third, the AD of the middle part of the cisternal trigeminal nerve, the FA of bilateral nuclear zones, and the MD of the center of PCT significantly changed over time before and after treatment. Fourth, the difference of GMV in the pain-matrix regions between the two hemispheres exhibited similar trends to the VAS before and after treatment. Finally, the DTI indices in the cisternal trigeminal nerve (FA) and nuclear zone (RD and MD) of the affected side were significantly correlated with the GMVs in specific pain-matrix regions (PAG, right S1, and left thalamus) before and after treatment. These findings are further discussed in the following parts of the document.

In the DTI analysis, the results showed that patients with TN exhibited slightly lower AD and FA values with no statistical significance in the affected side of the cisternal segment of the trigeminal nerve before treatment. The significant changes in the AD, RD, MD, and FA values in the affected side after treatment suggested that the treatments significantly altered the microstructural diffusion in the trigeminal nerve. In contrast, the REZ and nuclear zones yielded significant differences in the values of the DTI indices between the two sides before and after treatment, thus indicating that TN led to more tissue alterations in the REZ and nuclear zones than the cisternal trigeminal nerve, most likely owing to the fact that 67% (8/12) of studied patients exhibited NVC at REZ.

Our study also demonstrated that patients with TN not only had lower FA but also had higher AD, RD, and MD values at the affected REZ. Consistent with a prior study [24], our findings suggested that the increased MD and RD may be linked to NVC-induced focal demyelination in the trigeminal nerve REZ [25], neuroinflammatory processes, and/or edema [26] that affected the trigeminal system. Therefore, DTI can detect subtle pathological features at the trigeminal nerve REZ supporting a role for this regional involvement in TN pathophysiology. Moreover, the present study performed the treatments with a focus on the middle part of the cisternal trigeminal nerve in the affected side. Thus, the significant changes of the FA values in the distal and contralateral REZ and nuclear zone suggested that the Wallerian degeneration occurred through the PCT [27, 28], wherein minor changes of the DTI indices were observed after treatment.

The present study employed 3D T2-SPACE images to examine cortical/subcortical brain GMVs based on VBM analysis. A previous study reported that patients with TN had greater GMVs in the thalamus, contralateral S1, amygdala, frontal pole, PAG, primary motor cortex, and basal ganglia and cortical thinning in the orbitofrontal cortex, pregenual ACC, and insula [29], whereas anther study reported that patients with TN showed GMV reductions including the frontal, temporal, and parietal areas, as well as in the left thalamus and right cerebellum [30]. Our study found that patients with TN had significantly lower GMVs in PCC, ACC, insula, amygdala, and S1 in the affected side compared with the contralateral side before treatment. These findings may reflect unique symptoms because TN is characterized by paroxysmal pain triggered by innocuous stimuli and does not involve major sensory losses. However, the differences between the previous findings and ours may be attributed to the mixed trigeminal pain patient groups and the different methodology (T1-VBM versus T2-VBM) used to assess GM changes.

In addition, our results demonstrated that TN led to significant GMV differences between the two hemispheres in some pain-related brain regions (PCC, ACC, insula, amygdala, and S1) and that the therapy helped reduce the GMV difference at 1 month after treatment. However, the GMV differences in some pain-related regions (PCC, insula, amygdala, and S2) became significant again at 6 months after treatment but returned to insignificant outcomes at 12 months after treatment. The sequential changes of GMV in the pain-matrix regions were generally consistent with the VAS in patients with TN. Initially, the pain intensity was measured to be as high as 9.25 ± 0.97 before treatment and was significantly reduced to 0.08 ± 0.29 after 1 month of treatment. However, at 6 months after treatment, two patients who exhibited degraded pain intensity, which resulted in a slight increase in VAS (0.92 ± 2.61), received a second treatment. After the second treatment, one patient had improved symptoms with the VAS reduced from 9 to 2, but the other had deteriorated symptoms with a VAS increased from 2 to 4. As a result, the overall VAS was slightly decreased after 12 months of treatment. Moreover, the correlation analysis revealed significant correlations between the DTI indices of the cisternal trigeminal nerve and nuclear zone and the GMVs of the pain-matrix regions in the affected side before and after treatment. These findings suggested that the changes of GMV in the pain-matrix regions were likely associated with the changes of VAS and that VBM analysis of T2-SPACE was helpful in the noninvasive monitoring and reflection of the pain intensity in patients with TN before and after treatment.

The etiology and pathophysiological mechanisms of TN are still not well understood, and the central contributions in TN are still debated. The most common theory of the classical TN etiology is a peripheral theory that involves the compression of the trigeminal nerve's REZ by blood vessels owing to the surgical experience [5]. Moreover, several studies demonstrated pathological changes and most notably the demyelination of the trigeminal nerves in patients with TN [6, 31–35]. In the present study, despite the fact that minor changes were observed in the DTI indices in the cisternal segment of the trigeminal nerve, no statistical significance was revealed between the affected and contralateral sides before treatment. Nevertheless, the REZ and nuclear zone yielded significant changes in the DTI indices before treatment and more significant changes than the cisternal segment of trigeminal nerve after treatment. This suggested that the REZ and nuclear zone were more sensitive to TN before treatment and had more demyelination after treatment compared with the cisternal segment of the trigeminal nerve.

However, the NVC theory cannot sufficiently explain the etiology and pathophysiological mechanisms of TN because some individuals can develop TN without NVC, and some individuals with NVC never develop TN. In the present study, 25% (3/12) of the studied patients developed TN without NVC. This suggests that the TN symptoms were not fully attributable to the presence of NVC. It is known that peripheral nerve injury can lead to central nervous system (CNS) plasticity [36, 37] most likely owing to the sustained or repetitive activation of primary afferent fibers and central sensitization [38]. In the present study, our results demonstrated that the changes of DTI indices in the trigeminal nerve were associated with the alterations of GMVs in brain pain-matrix regions before and after treatment. Furthermore, the longitudinal changes of GMV in the pain-matrix regions were generally consistent with the sequential changes of VAS, thus suggesting that the CNS changes in conjunction with trigeminal nerve injury contribute to TN symptoms.

The study is associated with some limitations. First, the small sample size of this study may lead to a low statistical power. Thus, the results ought to be interpreted with care. Second, some patients did not undergo a follow-up MRI scan owing to refusal or dropout. Thus, the incomplete dataset may affect the statistical results. Third, comparisons were not conducted between patients with TN and healthy controls in DTI and VBM analysis because only four age- and sex-matched healthy controls were enrolled in this study. Further investigations with larger groups will be needed to comprehensively compare the differences of cisternal trigeminal nerve and brain pain-matrix regions among healthy subjects and patients with TN before and after treatment. Finally, although the RESOLVE DTI exhibited less susceptibility distortions, it has drawbacks of longer scan time and lower signal-to-noise ratio efficiency than single-shot DTI due to the nature of multi-shot acquisition and shorter readout time, respectively. Thus, the results of RESOLVE DTI may have been likely affected by motion artifacts.

## Conclusions

MRI with RESOLVE DTI was capable of detecting microstructural changes in patients with refractory TN before and after treatment, as demonstrated by the longitudinal changes of the FA, AD, RD, and MD values on the symptomatic side of the trigeminal nerve cisternal segment, REZ, nuclear zone, and PCT. The VBM analysis of T2-SPACE data was helpful in detecting longitudinal changes of GMV in the pain-matrix regions on the symptomatic cerebral hemisphere in the studied patients before and after treatment. Accordingly, we conclude that RESOLVE DTI and VBM analysis with T2-SPACE images were helpful in the understanding of the pathophysiological mechanisms in patients with TN before and after treatment.

## Data Availability

The data presented in this study are available on request from the corresponding author. The data are not publicly available due to the nature of this research, participants of this study did not agree for their data to be shared publicly.
